# Current trends in the allocation of National Institute of Health funding of brain tumor research

**DOI:** 10.1093/noajnl/vdae203

**Published:** 2024-12-04

**Authors:** Noah L A Nawabi, Brian F Saway, Rohan Jha, Matheus Pereira, Neel H Mehta, Arabinda Das, Alicia Zukas, Scott Lindhorst, Ben A Strickland

**Affiliations:** College of Medicine, Medical University of South Carolina, Charleston, South Carolina; Department of Neurosurgery, Medical University of South Carolina, Charleston, South Carolina; Harvard Medical School, Boston, Massachusetts; Department of Neurosurgery, Medical University of South Carolina, Charleston, South Carolina; Harvard Medical School, Boston, Massachusetts; Department of Neurosurgery, Medical University of South Carolina, Charleston, South Carolina; Hollings Cancer Center, Medical University of South Carolina, Charleston, South Carolina; Department of Neurosurgery, Medical University of South Carolina, Charleston, South Carolina; Hollings Cancer Center, Medical University of South Carolina, Charleston, South Carolina; Department of Neurosurgery, Medical University of South Carolina, Charleston, South Carolina; Hollings Cancer Center, Medical University of South Carolina, Charleston, South Carolina; Department of Neurosurgery, Medical University of South Carolina, Charleston, South Carolina

**Keywords:** brain tumor research, neuro-oncology, NIH funding

## Abstract

**Background:**

The National Institute of Health (NIH) provides a sizable annual budget toward brain tumor research. However, funding allocation for specific pathologies remains poorly described. We aimed to characterize the current landscape of NIH funding toward brain tumors as a function of pathology.

**Methods:**

NIHRePORTER was queried to identify studies focused on glioblastoma, pediatric glioma, oligodendroglioma, brain metastasis, meningioma, pituitary adenoma, and vestibular schwannoma, from 2000 to 2023. Studies with R, U, and P funding mechanisms were included. Data were compiled and assessed according to pathology.

**Results:**

Across these 7 tumors, 3320 unique studies with R, U, or P funding mechanisms were identified from 2000 to 2023. These were conducted across 480 unique institutions. The sum of funds allocated to all studies was $1 607 662 631. Glioblastoma commanded the largest portion of funds, representing 54% of R mechanisms, 55% of R01-funded studies, 48% of U mechanisms, and 49% of P mechanisms, and accounted for 51% ($813 556 423) of total funding. Brain metastasis was the second most-funded tumor, representing 31% of all R mechanisms, 31% of all R01-funded studies, 26% of all U mechanisms, and 28% of all P mechanisms, and accounted for 29% ($472 715 745) of funding. The remaining 14% of R mechanisms, 26% of U mechanisms, and 23% of P mechanisms focused on the remaining pathologies, and accounted for 20% ($321 390 463) of funding.

**Conclusions:**

The current landscape of NIH funding for brain tumor research indicates that awarded mechanisms prioritize malignant intra-axial malignancies. Despite their prevalence, skull base neoplasia is far less represented in NIH-funded studies.

## Lay Summary

The National Institutes of Health (NIH) allocates a large amount of funding each year to support brain tumor research. The authors of this study wanted to understand how the money is allocated to study the different types of brain tumors. To do this, they reviewed ~3300 research projects that were supported by the NIH from 2000 to 2023 and determined whether the funding was provided to study glioblastoma, brain metastases, meningioma, and/or other types of brain tumors. Their results showed that the NIH provided more than $1.6 billion in funding during this period. More than half of this amount ($813 million) went toward research projects on glioblastoma, the most aggressive brain cancer. Nearly, one-third ($473 million) was directed to research studies on brain metastases, which are cancers from elsewhere in the body that spread to the brain. Less aggressive brain tumors, such as meningiomas which are relatively common and can significantly affect the quality of life, received far less funding.

**Key Points**

- Totally $1 607 662 631 was allocated to neuro-oncology research by the National Institute of Health from 2000 to 2023.
- GBM (54%) and brain metastases (31%) were the 2 most-funded pathologies.
- Intra-axial malignancies significantly out-funded more prevalent skull-base neoplasia.

**Importance of the Study**

It has been well established that neuro-oncology research in the United States has become increasingly robust in recent years. However, the precise funding landscape within the realm of neuro-oncology research has not been well described. Trends in government funding for specific brain tumors and for neuro-oncology research have not been comprehensively assessed, to our knowledge. In the present study, we review the total funding made available to neuro-oncology research by the National Institutes of Health and assess at a granular level how funds were distributed to different pathologies. We also examine the most funded areas of biomedical research within each tumor subtype and highlight institutions that were the biggest stakeholders in this realm over the last several decades. This information is key to developing an understanding of how resources have been distributed among neuro-oncology research historically and may help address opportunities for growth in certain domains of brain tumor research.

There are ~200 000 new diagnoses of brain tumors each year in the United States.^1,2^ These neoplasms are associated with significant morbidity and mortality, with the ramifications reflected by the sizable cost posed to our healthcare system secondary to brain cancer admissions.^3^ Many of these pathologies are associated with poor prognoses. Necessarily, there are substantial resources dedicated to basic, translational, and clinical research efforts focused on brain tumor-related topics. A critical component of sustaining such efforts is funding availability and allocation.

The National Institute of Health (NIH) is one of the primary sources of funding for brain tumor research in the United States and has seen a growth in research funding from 20 billion in the early 2000s to over 47 billion dollars in 2023.^4,5^ Accordingly, the funds available to neuro-oncology studies have been substantially amplified over the last several decades, to a more significant extent than other areas of central nervous system-related research.^6–9^ While robust, the precise funding landscape of neuro-oncology research has not been well described.^6,7,9^ Details including the most studied and most funded pathologies, the most significant stakeholders in North American neuro-oncology research, among others, have yet to be elucidated. In the present study, we aimed to describe the current spectrum of NIH funding toward brain tumors as a function of pathology.

## Methods

### Database Description

The NIH Research Portfolio Online Reporting Tools (NIHRePORTER) is a federally funded database of biomedical research projects conducted at various institutions across the United States. It is maintained by the NIH and contains information relevant to project focuses, timeline, and funding, among others. In the present study, the NIHRePORTER website was queried to identify studies focused on the following brain tumors: glioblastoma, pediatric glioma, meningioma, oligodendroglioma, pituitary adenoma, vestibular schwannoma, and brain metastasis. While this list was not exhaustive, these pathologies were selected to capture the most prevalent tumors to provide insight into the funding trajectories of the most common intrinsic and extrinsic brain tumors. The present study did not require review by our Institutional Review Board given its observational nature and lack of patient inclusion.

### Study Identification

A simplified visualization of the study identification algorithm utilized in this study is illustrated in Supplementary Figure S1. For each tumor type, the name of the tumor as written above was pasted into the projects search bar on the NIHRePORTER website. For brain metastasis, both “brain metastasis” and “brain metastases” were searched individually, and their results were combined. “Astrocytoma” was not included as a search term due to the significant overlap present with the search results of “glioblastoma.” This yielded every ongoing and past project in the NIHRePORTER database relevant to each pathology. The “export” function was utilized to store search results locally in Microsoft Excel file format (.xlsx), and all available data fields were requested.

Once stored locally, the file containing one study per line was edited to display only the following study fields: “Project Title,” “Activity” (this field contained the funding mechanism), “Support Year,” “Project Start Date,” “Project End Date,” “Study Section,” “Contact PI/Project Leader,” “Department,” “Organization Name,” “Organization State,” “Budget Start Date,” “Budget End Date,” “Fiscal Year,” and “Total Cost.” According to the NIH, total cost is defined as “the total allowable costs (both direct costs and F&A costs) incurred by the recipient to carry out a grant-supported project or activity. Total project costs include costs charged to the NIH grant and costs borne by the recipient to satisfy a matching or cost-sharing requirement.”^10^ For our purposes, this variable was considered the total amount of funding dedicated to a project by the NIH.

Next, studies were removed from the analysis if they lacked a value for “Budget Start Date,” “Budget End Date,” or “Total Cost.” To isolate studies that started after January 1, 2000 and ended before January 1, 2024, studies that were funded in a fiscal year before 2000 or after 2023 were removed, as were studies with a funding start date that was pre-2000 or a funding end date that was post-2023. The “Activity” column was then sorted to remove all studies that had a funding mechanism other than R (Research Project Grants), U (Cooperative Agreements), or P (Program Projects). Finally, the “Project Title” column was filtered to remove duplicate values in order to isolate individual studies. This process was repeated for all 7 tumor types.

### Data Collection and Visualization

After isolating unique studies relevant to each of the described tumor types, project and tumor-specific data were collected. To identify total funding, the sum of the “Total Cost” column for each tumor type was calculated after removing duplicate projects. Average funding length was calculated by subtracting the funding start date from the funding end date and was then converted to months. The number of unique institutions per tumor type was collected by isolating values in the “Organization Name” column. The top-funded institutions for each tumor types were identified by calculating the highest total cost per organization. The same was done to identify the top-funded study sections per tumor type.

In order to demonstrate how funding over time changed for each tumor type, an Excel formula was utilized to calculate the amount of active funds per year. The total funding for each study was divided by the studies budget length (in months) to calculate funding per month for individual studies. For each month from January 2000 to December 2023, the amount of active funds per month was calculated by utilizing the funding per month and the project funding start and end dates for each study. To visualize the geographical distribution of top-funded institutions, Microsoft Excel and an online population mapping software (eSpatial) were utilized.^11^ Organization cities were isolated, and a similar formula used to calculate top-funded study sections was utilized to calculate the total amount of funding allocated to specific locations.

For each tumor type, number of unique studies, total R mechanisms and associated funding, total R01 studies, total P mechanisms and associated funding, and total U mechanisms and associated funding were also collected. Finally, top-funded academic institutions for each tumor type along with top-funded study sections were recorded.

All data manipulation was done in Microsoft Excel as previously described. Data visualization was conducted utilizing Microsoft Excel and GraphPad Prism.

## Results

**Figure 1.**
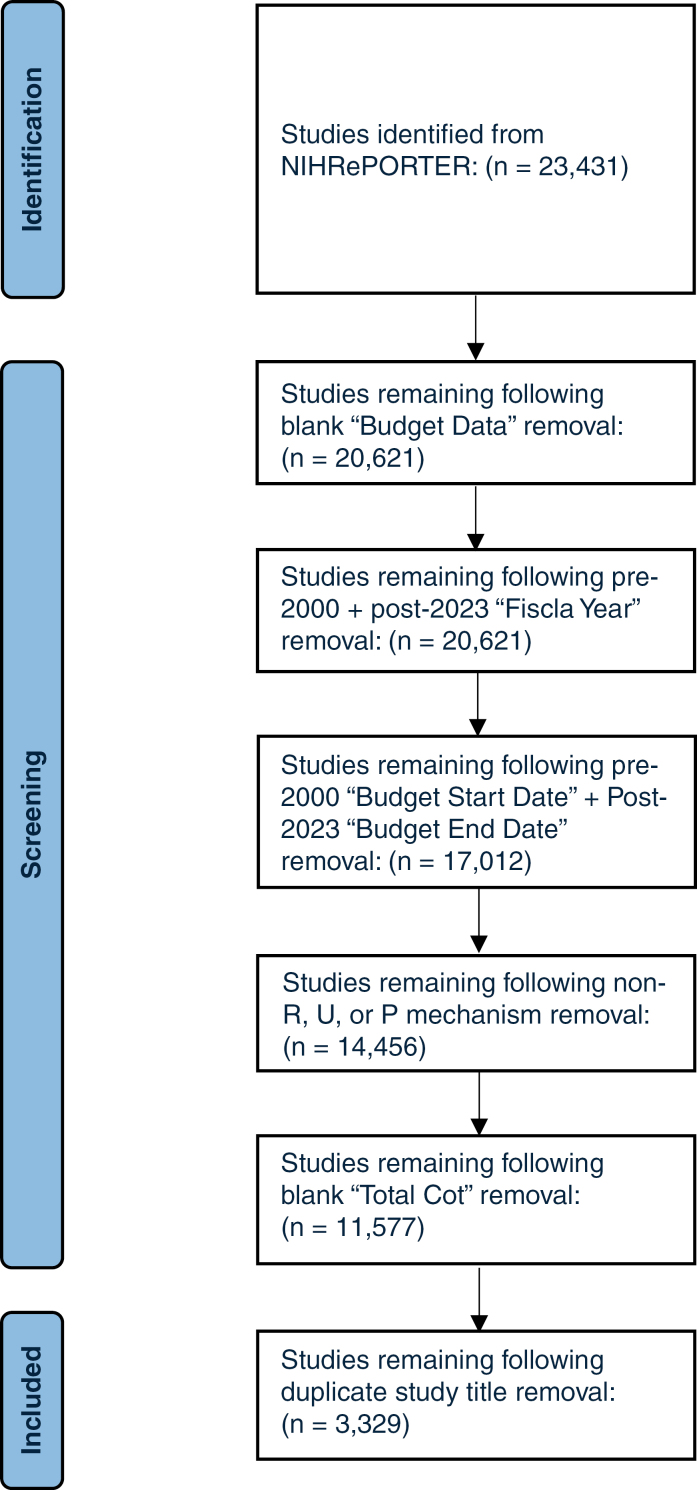
Flow chart demonstrating total studies remaining after each level of applying exclusion criteria.

A total of 23 431 studies were identified in NIHRePORTER pertaining to the 7 selected brain tumor pathologies. After exclusion criteria were applied, 3320 unique studies with R, U, or P mechanisms with funding dates between January 2000 and December 2023 were included (Figure 1).

**Figure 2.**
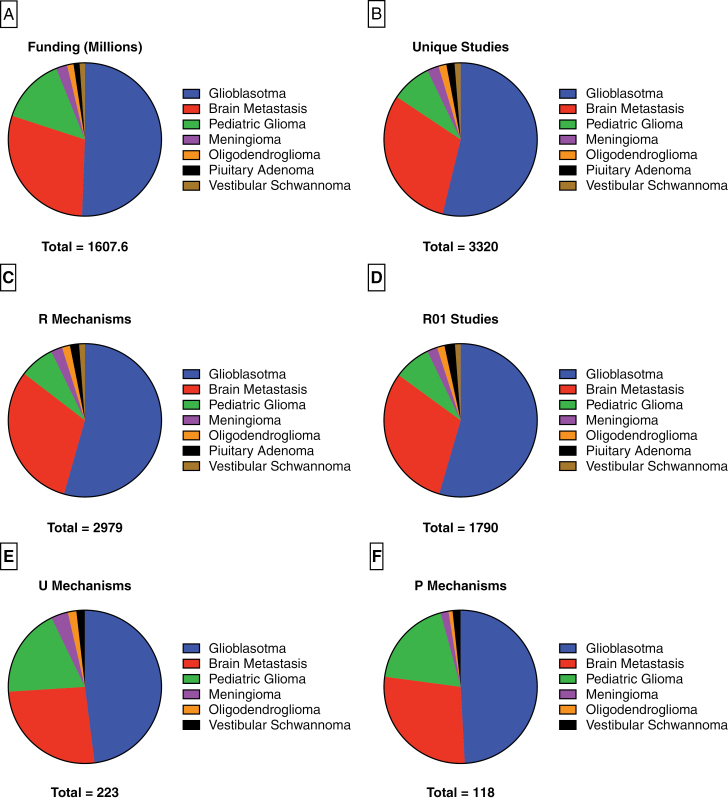
Pie charts demonstrating the distribution of unique studies (a), total funding (b), R mechanisms (c), U mechanisms (d), and P mechanisms (e) according to pathology.

Combined, these studies were allocated a total of $1 607 662 631 by the NIH. Glioblastoma commanded the vast majority of these funds, receiving $813 556 433 (51%) of NIH funding during the study period, which was more than all other pathologies combined. While a distant second, brain metastasis received $472 715 745 (29%) in funding, over twice as much as pediatric glioma, which was the third highest-funded tumor at $222 818 846 (14%). The remaining four tumors received a fraction of the funding allocated to these more aggressive pathologies. Meningioma was the highest funded of these at $38 333 480 (2%), followed by oligodendroglioma which received $21 728 792 (1%). Pituitary adenoma and vestibular schwannoma were the 2 lowest-funded tumors in the present study at $20 682 519 (1%) and $17 826 826 (1%), respectively (Figure 2a).

The distribution of study focus was aligned with that of total funding, with glioblastoma serving as the focus of 54% of all studies, followed by brain metastasis (31%). This held true regardless of the selected mechanism, with glioblastoma dominating the scope of R mechanisms (54%), R01 studies (55%), U mechanisms (48%), and P mechanisms (49%), with brain metastasis a clear second for all. While still the third most studied tumor, pediatric glioma commanded a smaller proportion of total studies (8%), than it did total funding (14%). Meningioma, oligodendroglioma, pituitary adenoma, and vestibular schwannoma were the focus of 2% or less of all studies when considered individually (Figure 2b-f). The precise funding amounts and mechanism distribution across all tumor types are shown in Table 1.

**Table 1.** Study and Funding Distribution by Pathology

Pathology	Total studies	Total funding	R Mechanisms	R01	U Mechanisms	P Mechanisms	Mean funding length (Months)
Total	3320	$1 607 662 631	2979	1790	223	118	15 (8)
Glioblastoma	1786 (54%)	$813 556 423 (51%)	1621 (54%)	976 (55%)	107 (48%)	58 (49%)	15 (8)
Brain Metastasis	1015 (31%)	$472 715 745 (29%)	924 (31%)	546 (31%)	58 (26%)	33 (28%)	16 (8)
Pediatric Glioma	282 (8%)	$222 818 846 (14%)	218 (7%)	140 (8%)	42 (19%)	22 (19%)	14 (7)
Meningioma	82 (2%)	$38 333 480 (2%)	72 (2%)	39 (2%)	8 (4%)	2 (2%)	15 (8)
Oligodendroglioma	55 (2%)	$21 728 792 (1%)	50 (2%)	28 (2%)	4 (2%)	1 (1%)	16 (7)
Pituitary Adenoma	58 (2%)	$20 682 519 (1%)	58 (2%)	40 (2%)	0 (0%)	0 (0%)	15 (7)
Vestibular Schwannoma	42 (1%)	$17 826 826 (1%)	36 (1%)	21 (1%)	4 (2%)	2 (2%)	14 (6)

**Figure 3.**
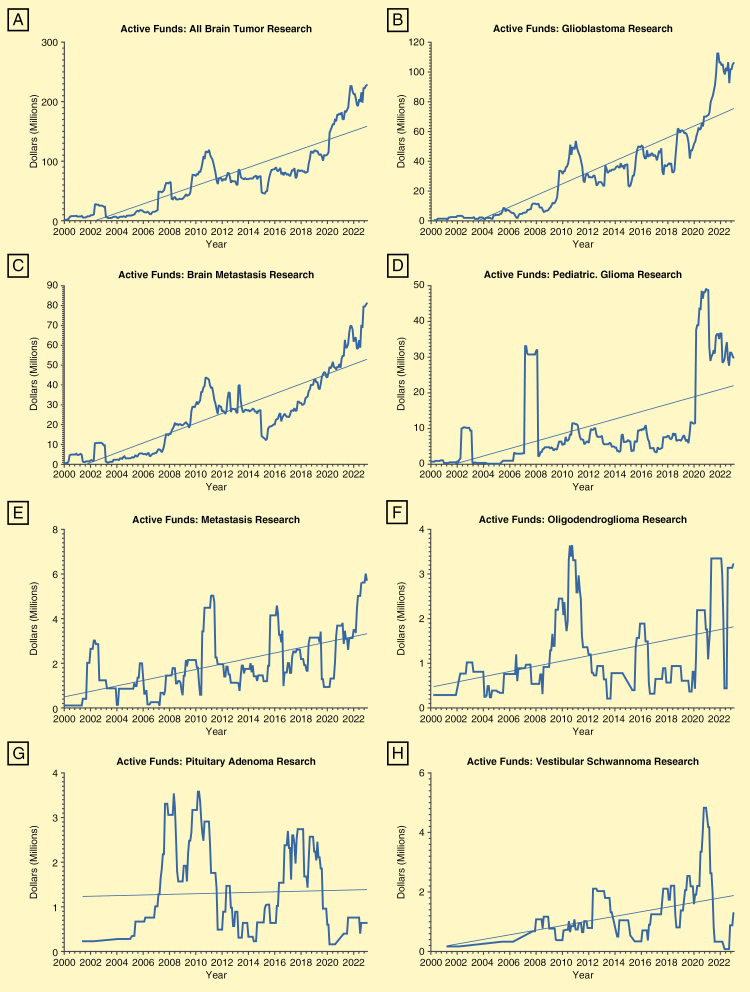
Active funds per month from January 2000 to December 2023 were calculated for each pathology utilizing the “Total Cost,” “Budget Start Date,” and “Budget End Date” columns. Active funds for all tumor research (a), glioblastoma (b), brain metastasis (c), pediatric glioma (d), meningioma (e), oligodendroglioma (f), pituitary adenoma (g), and vestibular schwannoma (i) are illustrated by the thicker line. Trend lines visualizing the change over time according to logistic regression are visualized by the thinner line.

Active funds per month for brain cancer research as a whole and for individual pathologies were calculated utilizing data on budget start and end dates for each project along with total cost. As seen in Figure 3, funding increased exponentially throughout the entirety of the study period for all tumors, both when considered together and individually. In January of 2000, a total of $1 442 986 were active across all studies, compared with $227 981 270 in January of 2023 (Figure 3a). This trend was largely dominated by glioblastoma funding (Figure 3b), although brain metastasis studies saw a similarly linear increase in funding over the past two decades (Figure 3c). The trends for funding of the remaining tumors were less linear, but allocated resources increased in absolute terms for all pathologies by the end of the study period, nevertheless (Figure 3d-h).

**Figure 4.**
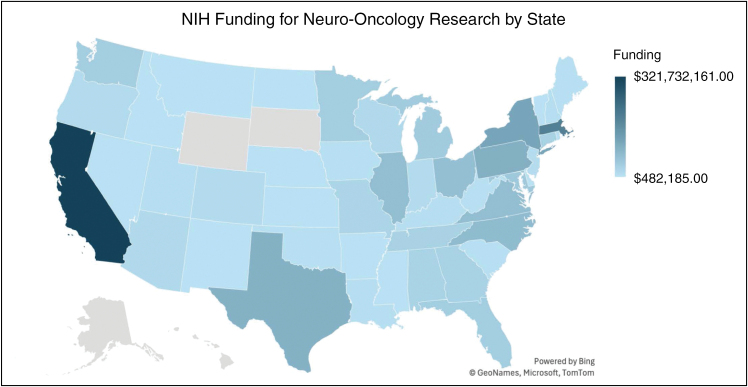
Geographic distribution of funds is visualized utilizing Microsoft Excel and the mapping software eSpatial. The total funding and state of each organization with neuro-oncology-focused R01 grants were utilized to illustrate the geographic spread of brain tumor research resources in the United States. Darker blue indicates a higher density of funding.

A total of 480 academic institutions were represented in the final body of included brain tumor projects. The geographic distribution of these institutions with associated funding levels is visualized in Figure 4. The most funded institutions across all tumors were the University of California, San Francisco (UCSF), Massachusetts General Hospital (MGH), and Duke University. MGH and UCSF were consistently ranked in the top 3 of funding for individual pathologies, with MGH being ranked in the top 3 for 6 of 7 pathologies and UCSF in 3, while Duke was ranked third in total funding for glioblastoma alone. Only 9 other unique institutions were ranked in the top 3 of any 1 tumor type (Table 2). The top-funded study sections, or the groups responsible for reviewing grant applications, across all pathologies were “Clinical Groups Study Section,” followed by “Clinical Neuroimmunology and Brain Tumors” and “Developmental Therapeutics.” “Clinical Neuroimmunology and Brain Tumors” was the top-funded section for both glioblastoma and oligodendroglioma, while “Clinical Groups” and “Tumor Progression and Metastasis” were the top-funded sections for pediatric glioma and brain metastasis, respectively. The complete rank list for study section funding can be found in Supplementary Table S1.

**Table 2.** Top-Funded Institutions by Pathology

Pathology	Total institutions with funding	Institution funding ranked		
		1st	2nd	3rd
Total	480	UCSF	MGH	Duke
Glioblastoma	335	MGH	UCSF	Duke
Brain Metastasis	294	MGH	Yale	UABB
Pediatric Glioma	108	CHP	UCSF	Stanford
Meningioma	51	MGH	Yale	Johns Hopkins
Oligodendroglioma	29	UCSF	Stanford	MGH
Pituitary Adenoma	38	UM	Thomas Jefferson	MGH
Vestibular Schwannoma	28	MGH	UCF	U Chicago

## Discussion

Over the past two decades, the NIH has consistently augmented funding allocations toward neuro-oncology research. Our query based on predefined search terms of the NIHRePORTER website identified over 3000 unique studies focused on various intracranial pathologies, which were collectively funded with over 1.6 billion dollars of the NIH’s research budget, representing approximately one-eight of 1% of the total NIH research budget from 2000 to 2023 (801.7 billion dollars).^5^ Unsurprisingly, malignant pathologies were awarded the vast majority of these assets, although allocations for all tumor types grew throughout the study period. This represents the first focused evaluation of the distribution of financial resources toward the study of individual brain tumors.

Our results indicate a prevailing theme toward NIH funding: award allocation prioritizes poor prognosis as opposed to prevalence. Glioblastoma was the focus of over 50% of all brain tumor studies and received over $800 000 000 (51%) of identified NIH funding. This is likely reflective of a combination of the poor prognosis and prevalence associated with this disease, as glioblastoma accounts for nearly 50% of all malignant central nervous system tumors, and carries a median survival of 12-15 months, with less than 5% of patients surviving more than 5 years.^12–14^ Brain metastasis and pediatric glioma were awarded the second and third highest amount of NIH funding throughout the study period, respectively, although they trailed glioblastoma by a significant margin. Of note is meningioma funding which only yields ~3% of funding mechanisms despite being the most common tumor of the central nervous system. Although typically displaying a benign course, the high-grade variants still present in nontrivial numbers and without any reliable nonsurgical or radiation treatment paradigm. Indeed, prognosis appears to drive funding prioritization shaping the funding of neuro-oncology research over the past several decades.^2,15^

The factors that drive funding for certain pathologies within healthcare research remain a topic of debate. Some note that resources are universally awarded to diseases affecting advantaged populations, despite being rare, while others argue that the distribution of funds varies greatly depending on the nature of the funding organization.^16,17^ As a government agency, it is widely accepted that the NIH is required to make funding decisions that maximize global and/or national social value, and is therefore heavily influenced by public opinion and patient advocacy groups.^16,17^ The case of pancreatic cancer research funding in the United States effectively illustrates how these factors can play a role in NIH resource allocation, and how funds may at times be associated with clear improvements in patient outcomes. Before the founding of the Pancreatic Cancer Action Network (PanCAN) in 1999, funding for pancreatic cancer research by the NIH’s National Cancer Institute (NCI) was 17.3 million dollars, and 5-year survival for this disease was only 4%. By 2021, funding for pancreatic cancer research had increased by 1075% to over 203 million dollars in 2021, and 5-year survival had improved to over 13%, largely due to the efforts of the PanCAN.^18^ Patient advocacy groups such as these have been well-documented influencers of the research agenda in the United States.^19^ Similar advocacy groups for glioblastoma have been in existence for decades, all with the overarching goal of increasing awareness and improving outcomes for those affected by this pathology.^20–23^ The attention garnered from these groups, coupled with glioblastoma’s status as National Institute of Health one of the most common and deadly brain tumors, has likely played a substantial role in the ability of researchers studying this pathology to acquire funding.^24^

Despite the trends observed with pancreatic cancer, improvements in outcomes are seemingly independent of increases in funding for certain pathologies, as has been the case with glioblastoma.^17^ While the present study identified vast increases in funding for glioblastoma research, little progress has been made with respect to enhancing survival for these patients in recent years. The has been nearly two decades since the publication of the most recent significant advancement in glioblastoma incorporating temozolomide into treatment protocols.^12,25^ In fact, there has been a small increase in mortality for adults over the age of 65 with glioblastoma from 2004 to 2018, according to some sources.^26–28^ However, dismal outcomes for glioblastoma have not deterred physicians, researchers, or patients from seeking novel therapies, highlighted by the continued increases in funding for the development of next-generation approaches to enhancing overall and progression-free survival. “Developmental Therapeutics” was the second most funded study section for glioblastoma research in the present study, and “Drug Discovery and Molecular Pharmacology” was the third. CAR-T cell therapies, immune checkpoint inhibitors, oncolytic viruses, cancer vaccines, and other immunotherapies have been the focus of numerous randomized controlled clinical trials that have aimed to overcome the substantial barriers associated with achieving curative treatment for glioblastoma.^29,30^ Several of these studies have had promising preclinical rationale, and others have been shown to offer meaningful survival benefits in isolated populations.^13,31,32^ While these efforts have largely struggled to significantly impact survival at scale, they are laying the foundation for future works that may significantly improve outcomes for these patients, if funding and resource allocation continues to increase. It is the consensus that substantial efforts in the form of international, multicenter studies aimed at enhancing our understanding of the clinical and pathological characteristics of glioblastoma will be key in improving outcomes for this disease.^13^

Trends in outcomes for other tumors have been more assuring, despite their funding levels remaining well below those of the malignant pathologies. In a study by Cioffi et al., which utilized data from the National Program of Cancer Registries Survival Analytic file, it was determined that for patients between the ages of 40 and 64 diagnosed with oligodendroglioma, case fatality rates fell from nearly 44% in 2004-2007 to 10% in 2013-2017.^27^ Interestingly, our data show that the highest peak in funding for this pathology occurred just before this time period. There were over 3.5 million dollars in active funds for oligodendroglioma research in mid 2011. Likewise, for patients aged 65 + with meningioma, case fatality rates fell from 77% in 2004-2007 to under 40% in 2013-2017.^27^ Our data indicate that funding increased a near-high just before the onset of this time period for this pathology. While high points in funding for both of these tumors were observed in parallel with significant improvements in outcomes (according to a single study), oligodendroglioma received the lowest total funding of any tumor in the present study, and total funds for meningioma research were dwarfed by those awarded to glioblastoma studies. This highlights the often nonlinear and complex relationship between active funds and outcome improvements in clinical research and further suggest that prognosis likely plays a significant role in the allocation of NIH funding of brain tumor research.^17,27^

Meningioma was the most funded skull base pathology in the present study and was the focus of over 80 individual NIH-funded projects from 2000 to 2023. Meningiomas are the most common primary intracranial tumor, but unlike glioblastoma, the benign subtypes are considered to be highly responsive to treatment.^33^ World Health Organization (WHO) Grade I meningiomas, which make up ~80% of all meningiomas, rarely recur following gross total resection.^34,35^ Unfortunately, atypical and anaplastic variants comprised over an estimated 20% of meningioma have a high lifetime risk of recurrence and are often treatment-refractory.^36,37^ In such cases, there is often tumor progression despite optimal treatment strategies, and there are no reliable chemotherapy or immunotherapy options currently available.^38^ Achieving disease freedom in nearly 8000 patients diagnosed with high-grade meningioma variants each year in the United States has been arduous, historically.^36^ Yet, the formidable challenges presented by these pathologies have been met with less than 2% of the funding made available to glioblastoma research over the past two decades, despite a similar incidence. Nevertheless, outcomes for meningiomas have continued to improve, generally.^7,27,39^ This is likely reflective of advancements in clinical treatment paradigms powered by widespread investigations into the genomic and epigenomic landscapes of meningioma. Since the early 2000s, meningioma studies have focused heavily on diagnostics, establishing outcomes for various treatment modalities including surgical resection and radiotherapy, and treatments for challenging tumors including those located in the skull base and those with a high WHO grade.^40,41^ Given scientific advancements in the past two decades allowing for accessible transcriptomic and methylation profiling, meningioma studies have shifted to focus heavily on establishing more relevant classifiers that better predict tumor behavior than the histopathologic-based WHO classifier.^42,43^ This shift from defining outcomes for established treatments toward harnessing the understanding of the genomic and epigenomic makeup of meningiomas has made these tumors a prime target for the development of alternate adjuvant treatment options and immunological therapies.^44^ As clinical trials aimed at assessing the efficacy of these treatments continue to progress, it is likely that funding for meningioma research will rise above the levels observed in the present study in the years to come.

It remains evident that enhancing survival and prognosis for those afflicted by brain tumors relies heavily on research efforts and their continued funding, especially for pathologies shown to be categorically underfunded. The clinical trial landscape for glioblastoma stands alone currently in terms of caliber, although trials targeting other pathologies, including meningioma, will likely continue to materialize as advancements in molecular techniques and genomic assessments continue to develop. Within neurosurgery, a field that is highly research-oriented from the stages of medical student to chairmanship, it is crucial that harnessing the ability to acquire funds for research endeavors be made a focal point of neurosurgical training and practice. Identifying ways to engage all members of the neurosurgical community in these efforts will be key to improving outcomes for currently underfunded tumors, as will identifying factors that contribute to the ability to leverage funding for the purposes of clinical investigations.

The present study has several limitations. Most notably, we focused solely on NIH funding and did not capture foundational grants, which can be sizeable and may have reflected alternate trends compared with those identified here. Our ability to comment on funding for brain tumors as a whole is partially limited. Furthermore, we did not offer any formal analyses focusing on determining the nature of the relationship between funding and outcomes for several of these pathologies. These should be addressed in future studies that attempt to comment on the relationship between brain tumor funding and outcomes. Finally, the WHO classification criteria for glioblastoma changed several times throughout the study period, which could not be controlled for when querying NIHRePORTER. However, this did not detract from our key findings that glioblastoma as a pathology dominated the funding landscape for brain tumor research over the last two decades, regardless of fluid WHO classifications.

## Conclusions

The current landscape of NIH funding for brain tumor research demonstrates that tumors with a poor prognosis are more heavily funded, despite lower incidence and prevalence in some cases. NIH funding for all brain tumors has increased dramatically over the last several decades.

## Supplementary Material

vdae203_suppl_Supplementary_File_S1

vdae203_suppl_Supplementary_File_S2

## Data Availability

Any data utilized to produce statistics or visuals in the present study may be made available to interested authors upon request of the corresponding author.
