# Supplementary material for: Current trends in the allocation of National Institute of Health funding of brain tumor research
Source: Neurooncol Adv. 2024 Dec 4;7(1):vdae203. doi: 10.1093/noajnl/vdae203 (PMC11969036; doi:10.1093/noajnl/vdae203)
Supplement: vdae203_suppl_Supplementary_File_S2 [file vdae203_suppl_Supplementary_File_S2.docx]

Supplementary Table 1: Top-Funded Study Sections by Pathology

| Pathology | Study Section Funding Ranked | | |
| --- | --- | --- | --- |
|  | 1st | 2nd | 3rd |
| Total | Clinical Groups | Clinical Neuroimmunology and Brain Tumors | Developmental Therapeutics |
| Glioblastoma | Clinical Neuroimmunology and Brain Tumors | Developmental Therapeutics | Drug Discovery and Molecular Pharmacology |
| Brain Metastasis | Tumor Progression and Metastasis | Clinical Groups | Tumor Microenvironment |
| Pediatric Glioma | Clinical Groups | Cancer Clinical Investigation | Clinical Neuroimmunology and Brain Tumors |
| Meningioma | Neurological Sciences and Disorders | Cancer Genetics | Drug Discovery and Molecular Pharmacology |
| Oligodendroglioma | Clinical Neuroimmunology and Brain Tumors | Cancer, Heart, and Sleep epidemiology | Cancer Biomarkers |
| Pituitary Adenoma | Integrative and Clinical Endocrinology and Reproduction | Molecular and Cellular Endocrinology | Genetics of Health and Disease |
| Vestibular Schwannoma | Epidemiology of Cancer | Developmental Therapeutics | Auditory System |
